# Comparison between in-hospital stroke and community-onset stroke treated with endovascular thrombectomy

**DOI:** 10.1371/journal.pone.0214883

**Published:** 2019-04-12

**Authors:** Min-Yi Lu, Chih-Hao Chen, Shin-Joe Yeh, Li-Kai Tsai, Chung-Wei Lee, Sung-Chun Tang, Jiann-Shing Jeng

**Affiliations:** 1 Department of Neurology, Taipei Medical University Hospital, Taipei, Taiwan; 2 Stroke Center and Department of Neurology, National Taiwan University Hospital, Taipei, Taiwan; 3 Graduate Institute of Epidemiology and Preventive Medicine, College of Public Health, National Taiwan University, Taipei, Taiwan; 4 Department of Medical Imaging, National Taiwan University Hospital, Taipei, Taiwan; Instituto Mexicano del Seguro Social (IMSS) HGZ 2, MEXICO

## Abstract

**Objective:**

In-hospital stroke (IHS) is an uncommon but serious medical emergency. Early recanalization through endovascular thrombectomy (EVT) may offer a vital therapeutic choice. This study compared the clinical features and outcomes between IHS and community-onset stroke (COS).

**Methods:**

From a single-center registry of 2813 patients with ischemic stroke, those who had received EVT for acute ischemic stroke were included and classified into the IHS and COS groups based on their stroke onset scenario. We compared the outcomes including successful recanalization, symptomatic intracranial hemorrhage, functional independence (modified Rankin Scale score, 0–2) at 90 days, and mortality between the two groups.

**Results:**

A total of 24 patients with IHS (mean age, 70 years; 54% men) and 105 patients with COS (mean age, 73 years; 47% men) were included. The most frequently reported reasons for admission in patients with IHS were cardiovascular and oncological diseases. The initial National Institutes of Health Stroke Scale (NIHSS) scores and main occluded vessels were similar between the two groups. Patients with IHS received a higher number of active malignancy diagnoses, were more likely to withhold antithrombotic agents, and exhibited higher prestroke functional dependency. The median onset-to-puncture time was 192 min in IHS and 217 min in COS (*P* = 0.15). The percentages of successful recanalization (79% vs 71%), symptomatic hemorrhage (0% vs 9%), functional independence (42% vs 40%), and mortality (17% vs 12%) were comparable between the two groups. After adjustment for covariates, initial NIHSS scores and successful recanalization were the most important predictors for functional independence at 90 days.

**Conclusions:**

Despite having disadvantages at baseline, patients with IHS could still benefit from timely EVT to achieve favorable outcomes. A well-designed acute stroke protocol tailored for IHS should be developed.

## Introduction

In-hospital stroke (IHS) is an uncommon but serious medical emergency. Between 6.5% and 15% of all strokes may occur in the hospital depending on whether population-based or hospital-based registries are considered [1–3]. Patients with IHS usually exhibit specific predisposing risk factors and conditions prone to development of stroke, such as ongoing cardiovascular disease and receipt of surgery or invasive procedures [2, 4]. Previous studies have demonstrated a considerable delay in evaluation and treatment of IHS and a unanimously worse functional outcome among patients with IHS [3–6]. Currently, endovascular thrombectomy (EVT) is the standard acute treatment for large vessel occlusion strokes, and appropriate imaging selection allows broader diagnosis for patients, further extending the time window for stroke therapy [7–9]. Because some patients with IHS may be ineligible for treatment with intravenous recombinant tissue plasminogen activator (rt-PA) due to comorbidities or contraindications, early recanalization through EVT becomes a vital therapeutic choice.

Here, we present our single-center experience of EVT treatment in patients with IHS and compare their clinical features, treatment workflow, and outcome with patients with community-onset stroke (COS).

## Methods

### Patient selection and evaluation

Data were obtained from the stroke registry at the National Taiwan University Hospital (NTUH), which was established in 1995 to study the etiologic factors, clinical courses, prognoses, and complications of stroke [10, 11]. The university affiliated medical center has a capacity of more than 2000 beds. The study was approved by the research ethics committee of the hospital. Written informed consent was obtained from all participants or a family member of the participant. All consecutive acute ischemic stroke cases between January 2015 and December 2017 were enrolled and divided into two groups. Patients were classified into the COS group if the onset of stroke occurred outside our hospital and they were sent to our hospital for management. A well-implemented stroke code was activated at the emergency department, and a consultant neurologist visited the patient to evaluate the eligibility for acute treatment such as rt-PA or EVT [12, 13]. Patients with IHS were defined as those who were staying in our hospital for more than 24 hours and exhibited new neurological deficits highly suspected to indicate acute stroke. The duty consultant neurologist, who is accessible 24 hours a day, visited the patient as soon as possible. The duty neurologist then arranged and coordinated further investigation and potential intervention for this acute stroke event. The protocol for management of IHS in NTUH is presented in S1 Fig.

For all patients, noncontrast brain computed tomography (CT) was arranged initially to exclude any intracranial hemorrhage (ICH). Stroke severity was evaluated using National Institutes of Health Stroke Scale (NIHSS) scores, which range from 0 to 42; the higher the score is, the greater the severity of the stroke is. If the patient fulfilled the eligibility criteria and had no absolute contraindications, rt-PA was administered within 4.5 hours from onset. However, if large artery occlusion stroke was suspected clinically (e.g., NIHSS ≥ 6, forced gaze deviation, or presence of cortical signs), brain CT angiography (CTA) and perfusion (CTP) studies were arranged immediately. The sites of occlusion and perfusion deficits were evaluated simultaneously by the duty neurologist and neurointerventionist. Generally, the criteria for EVT included the following: (1) groin puncture could be initiated within 6 h for anterior circulation stroke and within 24 h for posterior circulation stroke; (2) evidence of occlusion of the intracranial large artery such as distal internal carotid artery (ICA), first or second segment of the middle cerebral artery (M1 or M2 of MCA), first segment of the anterior cerebral artery, basilar artery, or intracranial vertebral artery; and (3) the existence of large ischemic mismatch/penumbra based on CTP for anterior circulation stroke. If EVT was indicated, the benefits and risks were explained to the patient and accompanying family member for their consideration. Of all patients with acute ischemic stroke, those who had received EVT were selected for the main analysis.

### Thrombectomy procedures

EVT was performed using any approved locally available administration device, such as a stent retriever, a thrombosuction device (Penumbra System), or both. Although most current evidence suggests the use of a stent retriever as the standard EVT device, the neurointerventionists in our hospital applied aspiration devices more frequently and were still able to achieve a similar recanalization success rate compared with previous major clinical trials [13]. Immediately after completion of EVT, a final angiography study was performed to assess the success of the procedure. A score of 2b or 3 in the modified Thrombolysis in Cerebral Infarction (mTICI) scoring system was defined as successful recanalization [14].

### Assessment of clinical and outcome variables

Demographic and clinical information, including age, sex, premorbid activities of daily living, NIHSS scores on admission, vascular risk factors (including hypertension, diabetes mellitus, hyperlipidemia, atrial fibrillation, coronary artery disease, family history of stroke, and previous stroke history), smoking or alcohol consumption, and relevant medication use (especially antiplatelet or anticoagulant medication use and any reason for temporally withholding such stroke-preventing medications) were collected. The subtype of stroke was further classified according to the Trial of Org 10172 in Acute Stroke Treatment criteria [15].

Premorbid functional independence was defined as a modified Rankin Scale (mRS) score of ≤1. The following time intervals in the thrombectomy procedures in the two groups were calculated and recorded: (1) onset to assessment, (2) onset to thrombolysis (if applicable), (3) onset to CTA, (4) onset to puncture, and (5) onset to first recanalization.

The objective of the study was to compare the functional outcome, reperfusion efficacy, and safety between patients in the IHS and COS groups. A favorable functional outcome was defined as an mRS of ≤2 in 90 days after stroke onset. Reperfusion efficacy was defined by successful recanalization (mTICI, 2b–3). Symptomatic ICH (sICH) was defined per SITS-MOST criteria as parenchymal hemorrhage type 2 combined with a neurologic deterioration of 4 points or more compared with baseline or the lowest NIHSS score within 24 hours [16].

### Statistical analysis

Continuous variables are expressed as mean ± SD or median (interquartile range) as appropriate, and categorical variables are expressed as proportions. Continuous variables were compared between the IHS and COS groups by using the Student’s *t* test or Mann–Whitney U test and categorical variables were compared using Chi-square or Fisher exact test when appropriate. Multivariable logistic regression analysis was applied to evaluate whether IHS was an independent predictor for a favorable functional outcome, and variables with statistical significance from the univariate analysis were entered as covariates. A *P* value of < 0.05 was considered significant. All data were analyzed using IBM SPSS Statistics for Windows.

## Results

From January 2015 to December 2017, a total of 2813 patients with acute ischemic stroke were admitted to the study hospital. Of them, 2477 (88.1%) were categorized into the COS group and 336 (11.9%) were categorized into the IHS group. Patients with IHS were more likely to have heart disease, dyslipidemia, and cancer; their initial stroke severity was higher than those with COS (Table 1). Moreover, they were more likely to have stroke-in-evolution, higher 1-month mortality, and worse 3-month functional status. Furthermore, the proportion of patients who received intravenous thrombolysis were significantly lower in the IHS group than in the COS group (3.6% vs 7.3%, *P* = 0.01).

**Table 1:** Demographics of in-hospital stroke and community-onset stroke.

	In-hospital stroke (n = 336)	Community-onset stroke (n = 2477)	*P* value
Age, years	65.9 ± 14.2	69.0 ± 13.9	<0.01
Male sex	206 (61.3)	1497 (60.4)	0.80
Admission NIHSS (median)	15 (6–25)	4 (2–10)	<0.01
Hypertension	207 (61.8)	1914 (77.3)	<0.01
Diabetes mellitus	136 (40.5)	989 (39.9)	0.89
Dyslipidemia	264 (78.6)	1339 (54.1)	<0.01
Coronary artery disease	96 (28.6)	339 (13.7)	<0.01
Atrial fibrillation	99 (29.5)	651 (26.3)	0.24
Previous stroke	48 (14.3)	466 (18.8)	0.05
Underlying malignancy	123 (36.6)	375 (15.1)	<0.01
Smoking habit	85 (25.3)	80 (0.54)	0.40
Ischemic stroke subtype			<0.01
Large artery atherosclerosis	26 (7.8)	434 (17.5)	
Small vessel occlusion	2 (0.6)	594 (24.0)	
Cardioembolism	149 (44.5)	667 (26.9)	
Other specific etiologies	47 (14.0)	146 (5.9)	
Undetermined etiology	111 (33.1)	636 (25.7)	
Stroke in evolution	191 (56.8)	470 (19.0)	<0.01
1-month mortality	95 (28.3)	112 (4.5)	<0.01
3-month mRS (median)	5 (3–6)	2 (1–4)	<0.01
IV thrombolysis only	6 (1.8)	136 (5.5)	<0.01
IV thrombolysis +/- EVT	12 (3.6)	182 (7.3)	0.01
EVT +/- IV thrombolysis	24 (7.1)	105 (4.2)	0.01

Values are number (percentage), mean ± standard deviation, or median (interquartile range).

During the study period, 129 consecutive patients had received EVT, of which, 24 were in the IHS group (mean age, 70 years; 54% men) and 105 were in the COS group (mean age, 73 years; 47% men). These figures suggested a higher chance of EVT receipt in patients with IHS than in those with COS (7.1% vs 4.2%; OR, 1.74 and 95% CI, 1.10–2.75) among all patients with acute ischemic stroke. The main reasons for admission of the patients with IHS were active cardiovascular disease (n = 9), survey or treatment of malignancy (n = 6), or preparation for surgical procedure (n = 4; Table 2). Active cardiovascular disease included myocardial infarction, arrhythmia (ventricular tachycardia, Brugada syndrome, and sick sinus syndrome), congestive heart failure, dilated cardiomyopathy, and cardiac valvulopathy. The type of malignancy included lung, breast, pancreatic, gallbladder, and unknown origin of metastasis. Surgical procedures included ophthalmology, orthopedics, and general surgery. Correspondingly, the location of stroke onset among the patients with IHS was mainly cardiovascular, oncological, or medical departments. Seven patients with IHS were under antithrombotic agents during the stroke event, whereas such treatment was withheld from four patients (all anticoagulants, two due to preparation for procedure and two due to suspicious bleeding complications).

**Table 2:** Characteristics of patients with in-hospital stroke.

**Patient location at time of stroke**	
Cardiology department	7
Oncological department	6
Other medical department	6
Other surgical department	4
Emergency department	1
**Reason for hospital admission**	
Active cardiovascular diseases	9
Survey or treatment of malignancy	6
Surgical procedure	4
Suspected stroke	3
Other medical reasons	2
**Concurrent use or withhold of antithrombotic agents**	
Use of antiplatelet agents	5
Use of vitamin K antagonists	2
Use of direct oral anticoagulant	1
Withhold of vitamin K antagonists	3
Withhold of direct oral anticoagulant	1

**Fig 1:**
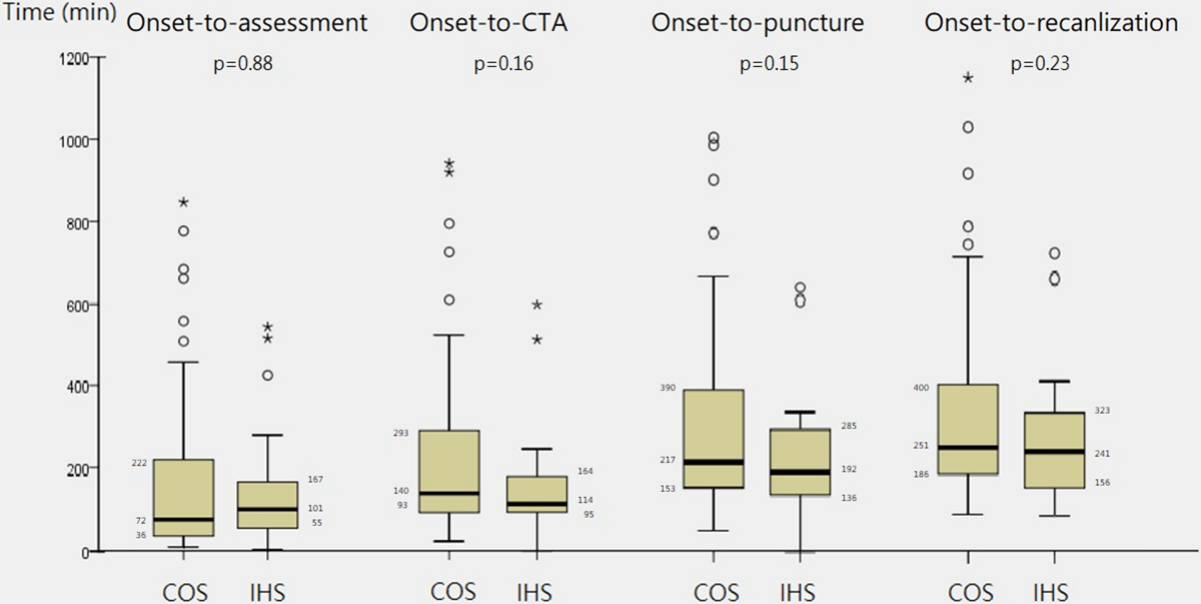
Comparison of the workflow time intervals of endovascular thrombectomy in patients with IHS and COS.

Overall, patients with IHS had higher prevalence of underlying cancer diagnosis (29% vs 9%, *P* = 0.01), were less likely to have atrial fibrillation (33% vs 68%, *P* < 0.01), were more likely to withhold antithrombotic agents (17% vs 3%, *P* = 0.03), and had less premorbid functional independence (58% vs 96%, *P* < 0.01) compared with patients with COS (Table 3). Regarding the stroke event, the median initial NIHSS scores (19 vs 19) were identical between the two groups, and the main occluded vessels were the ICA (29% vs 23%), M1 of MCA (61% vs 51%), and M2 of MCA (8% vs 13%). The proportion of bridging therapy with intravenous thrombolysis was slightly lower in the IHS group (29% vs 44%, *P* = 0.14). The time intervals of onset to neurologist’s assessment, intravenous thrombolysis, CTA, groin puncture, and first recanalization were comparable between the two groups, with a median onset-to-puncture time of 192 minutes in the IHS group and 217 minutes in the COS group (Fig 1). In addition, to explore whether the workflow within the hospital was similar between the two groups, we calculated the time intervals starting from neurological assessment. The results revealed that all time parameters were significantly shorter in the IHS group (S2 Fig). The predominant thrombectomy technique in all the patients was thrombosuction alone (60%) or thrombosuction combined with the use of a stent retriever (18%).

**Table 3:** Comparison between patients of in-hospital stroke and community-onset stroke receiving endovascular thrombectomy.

	In-hospital stroke (n = 24)	Community-onset stroke (n = 105)	*P* value
**Demographics**			
Age	70.1 ± 14.1	72.9 ± 12.2	0.33
Male	13 (54.2)	49 (46.7)	0.33
Baseline independence	14 (58.3)	101 (96.2)	<0.01
Hypertension	10 (41.7)	64 (60.9)	0.13
Diabetes mellitus	7 (29.2)	27 (22.9)	0.45
Dyslipidemia	5 (20.8)	32 (30.4)	0.25
Atrial fibrillation	8 (33.3)	71 (67.6)	<0.01
Previous stroke	5 (20.8)	28 (26.7)	0.38
Underlying malignancy	7 (29.2)	9 (8.6)	0.01
Smoking habit	2 (8.3)	6 (5.7)	0.46
Antiplatelet use	5 (20.8)	32 (30.4)	0.25
Anticoagulation use	2 (8.3)	12 (11.4)	0.50
Holding antithrombotics	4 (16.7)	3 (2.9)	0.02
**Stroke characteristics**			
Admission NIHSS (median)	19 (14–25)	19 (15–24)	0.78
Thrombolysis given	6 (25)	46 (43.8)	0.09
Main occluded vessel			0.35
Internal cerebral artery	7 (29)	24 (22.9)	
M1 of middle cerebral artery	15 (63)	53 (50.5)	
M2 of middle cerebral artery	2 (8)	14 (13.3)	
Anterior cerebral artery	0 (0)	1 (1.0)	
Vertebro-basilar artery	0 (0)	13 (12.4)	
Ischemic stroke subtype			0.01
Large artery atherosclerosis	2 (8.3)	14 (12.4)	
Small vessel occlusion	0 (0)	0 (0)	
Cardioembolism	16 (66.7)	80 (76.2)	
Other specific etiologies	5 (20.8)	3 (2.9)	
Undetermined etiology	1 (4.2)	8 (7.6)	
**Endovascular thrombectomy**			
Median time intervals			
Onset-to-assessment	101 (55–167)	76 (36–222)	0.84
Onset-to-CTA	114 (95–164)	140 (93–293)	0.15
Onset-to-thrombolysis	92 (77–125) ^a^	99 (71–125) ^a^	0.77
Onset-to-puncture	192 (136–285)	217 (153–390)	0.15
Onset-to-first recanalization	241(156–323)	251 (186–400)	0.25
Endovascular devices			0.90
Thrombosuction	13 (54.2)	65 (61.9)	
Stent retriever	2 (8.3)	6 (5.7)	
Combined use	5 (20.8)	18 (17.1)	
Others	4 (16.7)	16 (15.2)	
**Outcomes**			
Successful recanalization	19 (79.2)	75 (71.4)	0.31
Symptomatic ICH	0 (0)	9 (8.6)	0.14
Discharge NIHSS (median)	5 (2–25)	10 (2–19)	0.53
mRS at 90 days (median)	3 (1–5)	3 (1–4)	0.74
Good outcome (mRS 0–2)	10 (41.7)	42 (40.0)	0.53
Mortality	4 (16.7)	13 (12%)	0.58

Value are number (percentage), mean ± standard deviation, median (interquartile range). Abbreviation: CTA, computed tomography angiography; ICH, intracerebral hemorrhage; mRS, modified Rankin scale; NIHSS, National Institute of Health Stroke Scale

^a^ n = 6 in IHS and 38 in COS, respectively

**Fig 2:**
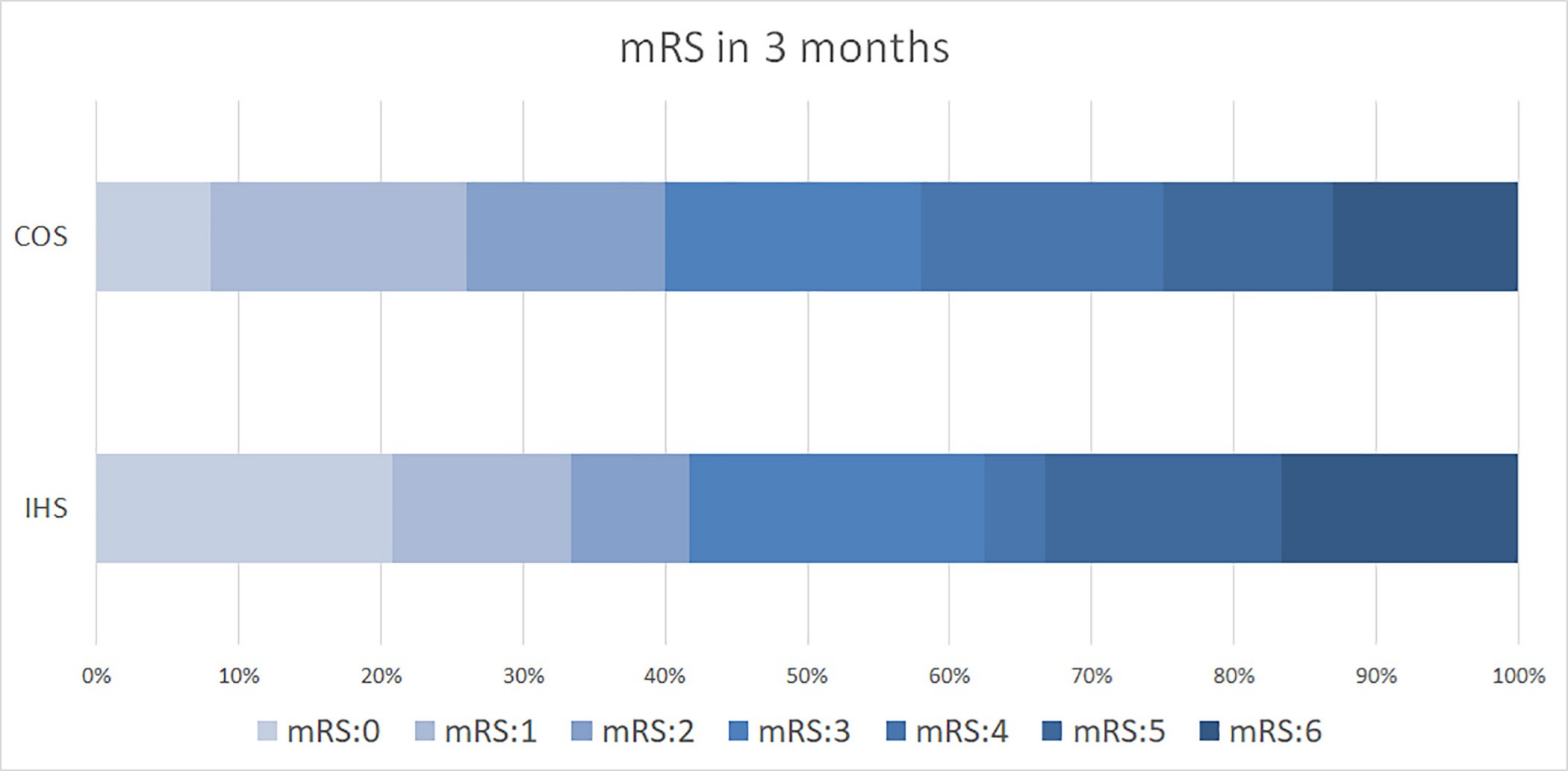
Functional outcome at 3 months evaluated by whole spectrum modified Rankin scale between patients with IHS and COS.

Regarding the efficacy and safety outcomes, the proportions of patients with successful recanalization (79% vs 71%, *P* = 0.31), with sICH (0% vs 9%, *P* = 0.14), with favorable functional outcomes (42% vs 40%, *P* = 0.53, Fig 2), and who died (17% vs 12%, *P* = 0.58) at 90 days were comparable between the two groups. The median score of the mRS at 90 days was 3 in both the groups.

Univariate analysis showed that age, history of diabetes mellitus, previous stroke, admission NIHSS score, onset-to-puncture time, use of intravenous thrombolysis, and successful recanalization were the factors associated with a favorable functional outcome (Table 4). After multivariable adjustment, only successful recanalization, admission NIHSS score, and history of diabetes mellitus were found to be independent predictors for favorable outcomes. IHS itself was not an independent predictor in both unadjusted and adjusted models.

**Table 4:** Factors associated with a good functional outcome.

	Unadjusted OR (95% CI)	Adjusted OR(95% CI)
Age, year	0.96 (0.93–0.99) ^a^	0.96 (0.93–1.01)
Male gender	0.68 (0.33–1.37)	-
In-hospital stroke	1.07 (0.44–2.64)	1.24 (0.29–5.37)
Admission NIHSS	0.92 (0.86–0.97) ^a^	0.89 (0.81–0.97) ^a^
Thrombolysis use	2.23 (1.13–4.42) ^a^	1.32 (0.51–3.41)
Onset to puncture time (every 15 minutes)	0.96 (0.93–0.99) ^a^	0.97 (0.93–1.01)
Successful recanalization	11.6 (3.33–40.6) ^a^	15.8 (3.72–67.4) ^a^
Baseline independence	4.62 (0.99–21.6)	2.33 (0.29–18.9)
Hypertension	0.56 (0.28–1.13)	-
Diabetes mellitus	0.29 (0.11–0.73) ^a^	0.14 (0.04–0.50) ^a^
Hyperlipidemia	1.19 (0.55–2.57)	-
Atrial fibrillation	1.17 (0.57–2.42)	-
Previous stroke	0.31 (0.12–0.77) ^a^	0.44 (0.13–1.45)
Underlying malignancy	0.64 (0.21–1.96)	-
Smoking	0.78 (0.37–1.63)	-
Holding antithrombotics	2.06 (0.44–9.59)	-

^a^ indicating *P* value <0.05

## Discussion

The present study found that, relative to patients with COS, in a hospital equipped with a well-designed protocol and trained personnel, patients with IHS treated with EVT can achieve similar workflow efficiency and comparable proportion of favorable outcomes. This finding was encouraging because most previous studies have suggested that patients with IHS had worse functional dependency [3–6], which was usually attributed to poor baseline function and delay in diagnosis and timely intervention. However, previous studies have included all IHS patients irrespective of whether they received acute recanalization treatment. Our study provided unequivocal evidence that when recanalization, the current most effective strategy, was indicated and accessible, the patients with IHS could still benefit substantially from it.

Patients with IHS usually had more comorbidities and may undergo a certain degree of invasive procedures. These medical or surgical contraindications rendered them less likely to receive intravenous thrombolysis even with early presentation of symptoms. Our study proved this hypothesis by showing that the probability of receiving IV thrombolysis was only half in the IHS group compared with the COS group (3.6% vs 7.3%, OR = 0.47). Another multicenter registry in Canada also showed a lower use of thrombolysis in patients with IHS (12% vs 19%) [4]. Because mechanical thrombectomy became the class I recommendation for patients who met prespecified criteria [9] and the contraindications of EVT were less sophisticated than those of IV thrombolysis, patients with IHS were expected to benefit more from this newer treatment strategy. The present study demonstrated that among all patients with acute ischemic stroke, those with IHS had 70% higher chance of receiving EVT (odds ratio, 1.7) than those with COS.

In addition, the reasons for hospital admission in patients with IHS might render them more susceptible to intracranial major artery occlusion, which is the main indication for EVT in acute ischemic stroke [17]. For example, patients admitted to the cardiovascular department with a diagnosis of cardiovascular conditions such as arrhythmia, cardiomyopathy, and valvular heart disease exhibited a high risk of cardioembolism. Patients with active malignancy had a risk of hypercoagulable states, which in turn could result in multiple embolic stroke and potential major artery occlusion [18]. Discontinuation of antithrombotic agents in patients preparing to receive an invasive procedure or operation could also result in the possibility of ischemic stroke [19].

According to the literature, patients with IHS generally experienced a substantial delay in stroke symptom recognition, neurological evaluation, and appropriate intervention [2, 4, 6, 20]. This phenomenon might be related to the complexity of the patients’ underlying illness and hospital practice. However, these findings might be somehow misleading because not all patients with IHS were candidates for acute intervention. When only patients who had received acute intervention were considered, one study found an even faster onset to intravenous thrombolysis time among patients with IHS than among those with COS (128 vs 145 min, *P* < 0.001) [21]. Another study also demonstrated an improvement in assessment and treatment time after an IHS alert protocol was established in a single hospital [22]. In our study, the time intervals of onset to neurological assessment, neuroimaging, intravenous thrombolysis, groin puncture, and first recanalization were all comparable between the two groups. The time intervals were even shorter if only the postneurological assessment workflow was considered. However, this might still imply a potential delay between symptom onset to neurological assessment, and the most likely reason was lack of prompt recognition of stroke by caregivers. Because we had previously established a “stroke code” protocol for stroke patients to receive potential acute intervention [12], our neurological consultation team was already familiar with the workflow and also applied it in the management of IHS (S1 Fig). By using such a well-implemented protocol, more time could be saved in treating patients with IHS.

A faster reperfusion time is usually associated with more favorable outcomes in acute ischemic stroke [23, 24]. In our study, onset-to-puncture time was a borderline significant predictor for functional independence at 90 days. Because the onset-to-puncture time and onset-to-first-recanalization time were not different between the IHS and COS groups, the functional outcomes were also comparable. Other outcome indicators such as sICH, NIHSS score at discharge, median mRS score at 90 days, and mortality were all comparable between the IHS and COS groups. These findings suggested that IHS was not a poor prognostic factor by itself if a recanalization procedure could be performed.

To our knowledge, this is the latest study comparing patients with IHS and COS, especially focusing on those who received EVT. Although retrospectively analyzed, data were obtained from a prospectively collected registry that covered all consecutive stroke patients with approximately 1000 patients per year in one of the largest comprehensive stroke center in Taiwan [10]. The overall coverage and quality of the data was also satisfactory, with few missing items. However, some study limitations should be discussed. First, the study was conducted in a university-affiliated medical center. Whether our protocol based on abundant manpower, 24-hour on-call neurologists, and multidisciplinary teamwork could be successfully implemented in local or smaller hospitals is unclear. Second, we did not analyze the time intervals of the workflow in all IHS cases. This might have created a certain level of selection bias because patients who did not receive EVT may receive more prolonged and delayed treatment. However, our study aim was to examine the effects of EVT, and the results confirmed the internal validity. Third, we did not further classify patients with IHS based on their reasons of admission due to the small sample size. A larger sample size based on a multicenter registry might help to clarify whether the outcomes are different on receipt of recanalization.

## Conclusion

The present study demonstrated that despite having disadvantages at baseline condition, patients with IHS could still benefit from timely EVT. A well-designed acute stroke protocol tailored for IHS should be developed in each hospital for more favorable patient outcomes.

## Supporting information

S1 Fig Protocol for management of in-hospital stroke in National Taiwan University Hospital. (DOCX)

S2 Fig Comparison of the workflow time intervals of endovascular thrombectomy from neurologist’s initial assessment. (DOCX)

S1 Dataset (XLSX)
