# Supplementary material for: Comparison between in-hospital stroke and community-onset stroke treated with endovascular thrombectomy
Source: PLoS One. 2019 Apr 12;14(4):e0214883. doi: 10.1371/journal.pone.0214883 (PMC6461247; doi:10.1371/journal.pone.0214883)

**ONLINE SUPPLEMENT**

**Comparison between in-hospital stroke and community-onset stroke treated with endovascular thrombectomy**

Min-Yi Lu^1¶^, Chih-Hao Chen^2,3¶^, Shin-Joe Yeh^2^, Li-Kai Tsai^2^, Chung-Wei Lee^4^, Sung-Chun Tang^2*^, Jiann-Shing Jeng^2^

^1^ Department of Neurology, Taipei Medical University Hospital, Taipei, Taiwan

^2^ Stroke Center and Department of Neurology, National Taiwan University Hospital, Taipei, Taiwan

^3^ Graduate Institute of Epidemiology and Preventive Medicine, College of Public Health, National Taiwan University, Taipei; Taiwan

^4^ Department of Medical Imaging, National Taiwan University Hospital, Taipei, Taiwan

^*^ Corresponding author: Sung-Chun Tang

E-mail: [sctang@ntuh.gov.tw](mailto:sctang@ntuh.gov.tw) (SCT)

^¶^ *Drs Lu and Chen contributed equally.

**Corresponding author**: Sung-Chun Tang, MD, PhD. Department of Neurology, National Taiwan University Hospital, No. 7, Chung-Shan South Road, Taipei 10055, Taiwan.

E-mail: sctang@ntuh.gov.tw. Tel: 886-2-23562144; Fax: 886-2-23418395

**Supplemental Figure 2.** Comparison of the workflow time intervals of endovascular thrombectomy in patients with community-onset stroke and in-hospital stroke, based on the initial assessment time.


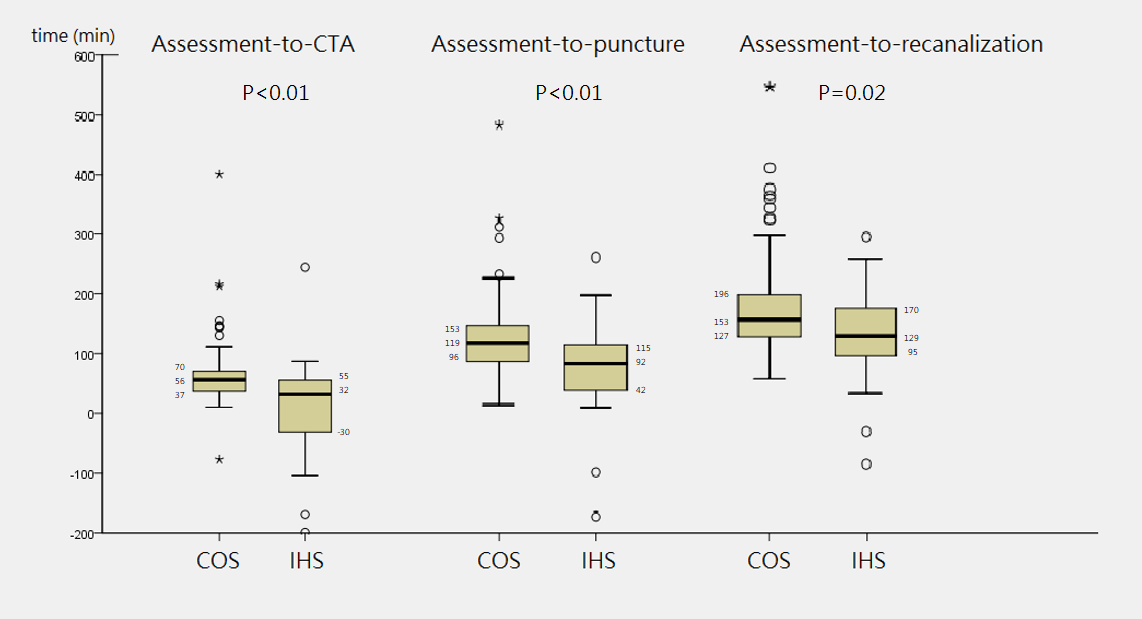

Supplement: S2 Fig — (DOCX) [file pone.0214883.s002.docx]
